# *Aerococcus urinae*, a cause of cystitis with malodorous urine in a child: clinical and microbiological challenges

**DOI:** 10.1099/jmmcr.0.005083

**Published:** 2017-02-28

**Authors:** Tilemachos Skalidis, Josef Papaparaskevas, Dimitrios Konstantinou, Eleni Kapolou, Mathhew E. Falagas, Nicholas Legakis

**Affiliations:** ^1^​ Central Laboratories, IASO Gynecology, Maternity and Pediatric Hospital, Iaso Group Hospitals, Athens, Greece; ^2^​ Department of Microbiology, Medical School, National and Kapodistrian University of Athens, Goudi, Athens, Greece; ^3^​ Neonatal Intensive Care Unit, IASO Paediatric Hospital, Maroussi, Athens, Greece; ^4^​ Department of Medicine - Infectious Diseases, IASO Group Hospitals, Athens, Greece

**Keywords:** *Aerococcus urinae*, cystitis, malodorous urine, bladder diverticulum

## Abstract

**Introduction.** An infection of the lower urinary tract associated with an extremely unpleasant odour due to *Aerococcus urinae* in an otherwise healthy 5-year-old boy is described herein.

**Case presentation.** Interestingly, imaging examination revealed the presence of a bladder diverticulum. Routine microbiological examination based on Gram staining, colony morphology and catalase reactivity suggested that the responsible pathogen could belong either to staphylococci, α-haemolytic streptococci or enterococci, which are more common urine isolates. Of note is that the VITEK 2 automated system could not identify the micro-organism. Susceptibility testing showed full sensitivity to β-lactam antibiotics and resistance to trimethoprim/sulfamethoxazole. The isolate was subjected to 16S rRNA gene sequence analysis because of its unusual characteristics. It was identified as *A. urinae* and the sequence was deposited in GenBank under the accession number KU207150.

**Conclusion.** *A. urinae* should be considered as a causative agent of urinary-tract infection associated with malodorous urine.

## Abbreviation

SXT, trimethoprim/sulfamethoxazole.

## Introduction

In paediatric patients, only four cases of urinary-tract infection or colonization have been reported in the literarture in the last few years. Herein, we report bladder diverticulum as a predisposing factor for *Aerococcus* *urinae* cystitis in a child.

## Case Report

A 5-year-old boy was referred to the paediatric outpatient clinic of IASO children's hospital for an unusual infection with foul-smelling urine for the previous 20 days. His parents reported that whenever the boy urinated, an extremely unpleasant smell spread. Interestingly, this situation was not associated with any other symptoms suggestive of urinary-tract infection, such as dysuria, or increased urination frequency or urgency. Fever was also absent. His previous medical history was unremarkable, and the physical examination revealed no pathological signs. Several urine samples examined previously in other settings showed an elevated number of leukocytes (50–70 per optic field, normal range 0–5 per optic field) and upon culture the growth of coagulase-negative staphylococci was reported, in numbers exceeding 10^5^ c.f.u. ml^−1^.

At the Central Laboratories of IASO Gynecology, Maternity and Pediatric Hospital, Greece, a urine examination showed an increased number of urinary leukocytes, while culture showed a single yield of Gram-positive cocci (>10^5^ c.f.u. ml^−1^) with colony morphology suggestive of α-haemolytic streptococci. Of note, no other bacteria grew. The bacterium was catalase negative and on Gram stain appeared in clusters resembling staphylococci in morphology. It was further investigated using the VITEK 2 system. However, neither identification nor antibiotic susceptibility could be obtained, as the system did not recognize the bacterium.

Because of the unusual characteristics of the urinary infection, efforts were made to elucidate the nature of the pathogen involved by sequencing of the 16S rRNA, considered the gold standard for species identification [1], carried out according to a sequencing protocol described elsewhere [2, 3]. The consensus sequence results obtained were compared with sequences available in GenBank using the blast algorithm. The isolate was identified as *A. urinae*. The respective sequence has been deposited in GenBank under the accession number KU207150.

**Fig. 1.**
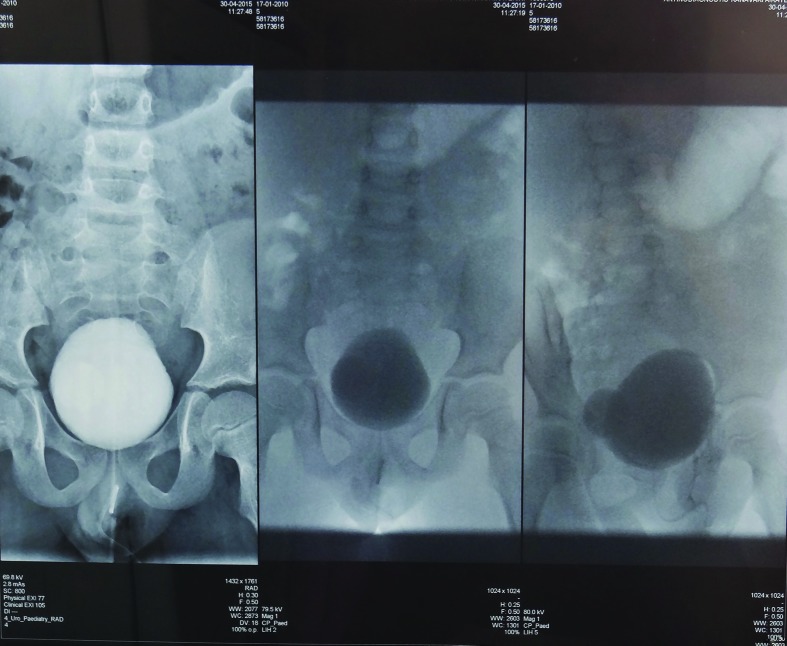
Bladder diverticulum.

Antimicrobial-susceptibility testing was performed at the time of isolation according to Clinical and Laboratory Standards Institute guidelines using the interpretive criteria for viridans group *Streptococcus* spp. as described elsewhere [4, 5]. The isolate showed susceptibility to all β-lactams, but resistance to gentamicin and trimethoprim/sulfamethoxazole (SXT). The child was treated with amoxicillin/clavulanate at a dose of 25 mg kg^−1^ per day, divided into three doses. After 3 days, the abnormal urine odour disappeared completely, and no leukocytes could be seen in the urine sediment. However, antibiotic administration continued for another 10 days, and 3 days after cessation of the treatment urine culture was found to be sterile. In investigating for underlying conditions predisposing the patient to the infection, the boy was examined with ultrasound and ascending urethrocystography, both of which revealed the presence of a bladder diverticulum 1.8 cm in diameter (Fig. 1). No other findings, e.g. solid masses, stones in the bladder or in the diverticulum, were noted.

## Discussion

Aerococci have been regarded until recently as rare causes of infections. The underestimation of the clinical impact of this pathogen was mainly due to the fact that these facultative anaerobic, catalase-negative, Gram-positive cocci have a colony morphology on culture similar to that of α-haemolytic streptococci, while on Gram stain they appear in clusters like staphylococci [6]. In addition, aerococci share some features of antibiotic susceptibility with enterococci in that they exhibit intrinsic resistance to SXT [7] and nalidixic acid.

Most case reports until recently demonstrated the clinical significance of *A. urinae* mainly as a causative agent of urinary infections in elderly patients with underlying urological pathology. Rare reports refer to *A. urinae* as a cause of invasive human infections, such as sepsis, endocarditis, peritonitis, spondylodiscitis and vertebral osteomyelitis [8–11]. In paediatric patients only one case of urinary-tract infection, a pyelonephritis not associated with malodorous urine [12], and three cases of colonization characterized by foul-smelling urine, have been reported in the last few years [13–15]. Interestingly, all the paediatric patients, including our own patient, were boys, in contrast to the elderly population where there seems to be equal gender distribution between men and women with aerococcal bacteriuria [16]. The gender predilection in children is interesting and needs further investigation. Clonal analysis of *A. urinae* by using repetitive extragenic palindromic PCR did not reveal any clonal relation among strains isolated from various clinical materials [17].

In our case, the bladder diverticulum was most probably the predisposing factor, as it occurs with respective infections in the elderly, although no malformations were present in the previously described cases [13–15], with the exception of the patient in the first case [12] who had a surgery for vesicoureteral reflux earlier in life. Our finding suggests that in cases of infection with *A. urinae*, a detailed morphological examination of the urinary tract should be considered.

The results of susceptibility testing using Mueller–Hinton Agar (MHA) supplemented with 5 % blood, showed resistance to SXT. However, other studies [18] using the broth dilution method and cation-adjusted MHA supplemented with 2.5 % lysed horse blood showed that most isolates were susceptible to SXT. Of note, laboratories may consider reporting *A. urinae* as SXT-resistant given that susceptibility to SXT *in vivo* may be dependent on the patient’s urinary folate concentration, which can vary considerably [18].

Several pathogenic bacteria infecting the urinary tract are able to produce various volatile metabolites, including trimethylamine, which could explain the characteristic malodorous urine. However, malodorous urine may have a wide aetiology, besides urinary colonization or infection. Drugs, metabolic disorders, incontinence, poor hygiene or hyperalimentation, with choline or l-carnitine containing foods, can produce the above phenomenon [19]. Paediatricians in such cases usually suspect a metabolic disorder and in particular trimethylaminuria (fish odour syndrome), which causes a similar foul-smelling urine as the leading symptom. Due to the wide antibiotic susceptibility of *A. urinae*, this pathogen should be excluded as a causative agent of urinary infection associated with considerably malodorous urine, before the initiation of any metabolic-disorder investigation.
